# A case-controlled study of minimally invasive vs open hallux valgus surgery

**DOI:** 10.1186/1757-1146-8-S1-A14

**Published:** 2015-04-20

**Authors:** Sarah Curran, Ashok Marudanayagam, Laura Beddard, Anthony Perera

**Affiliations:** 1 Department of Healthcare, Cardiff Metropolitan University, Cardiff, UK; 2 Cardiff and Vale University Health Board University Health Board, Cardiff, UK; 3 Spire Cardiff Hospital, Cardiff, UK; 4 London Foot and Ankle Centre, London, UK

## Background

Previous attempts at small incision hallux valgus surgery have compromised the principles of bunion correction in order to minimise the incision. The Minimally Invasive Chevron/ Akin (MICA) is a technique that enables an open modified Chevron/ Akin to be done through a 3mm incision, facilitated by a 2mm Shannon burr.

## Methodology

This is a consecutive case series performed between 2009 and 2012. This includes the learning curve for minimally invasive surgery. All cases were performed by a single surgeon at two different sites, one centre where minimally invasive surgery is available and the other where it is not. The standard procedure in both centres is a modified Chevron osteotomy. Regardless of whether the osteotomy was performed open or minimally invasive two-screw fixation was performed.

Retrospective analysis includes the intermetatarsal angle (IMA), hallux valgus angle (HVA), metatarsal 1 (M1) length, forefoot width and forefoot: hindfoot ratio. Clinical outcomes include the Manchester Oxford Foot Questionnaire (MOXFQ), American Orthopaedic Foot and Ankle Surgeons (AOFAS) questionniare, and assessment of complications.

## Results

There were 70 cases in each arm. Follow-up was 4 years to 6 months. The radiological outcomes were similar in both groups. There was an increased rate of screw removal in the MICA group. There were also cases of hallux varus, these occurred in the cases with severe pre-operative IMA angles that also had a lateral release and an Akin. There was high satisfaction in both groups.

## Conclusion

This is the only comparison of minimally invasive and open techniques that has been performed, providing a direct comparison of the utility of a burr compared to a saw. These early results demonstrate the efficacy of a Minimally Invasive Chevron/ Akin in terms of achieving radiological correction. The clinical outcomes are excellent but there is a learning curve and this needs to be managed.

